# To explore the protective mechanism of promethazine against hippocampal neuron injury based on network pharmacology and experimental verification

**DOI:** 10.1097/MD.0000000000040550

**Published:** 2024-12-06

**Authors:** Li Bai, Fang Li

**Affiliations:** a The First Affiliated Hospital of Jinzhou Medical University, Jinzhou, China; b Bazhong Central Hospital, Bazhong, China.

**Keywords:** Alzheimer disease, hippocampal neurons, oxidative stress, promethazine

## Abstract

This study aims to investigate the effect of promethazine (PMZ) on hippocampal neuronal injury through network pharmacology and in vivo experiments. Network pharmacology: The intersection genes of PMZ and Alzheimer Disease (AD) were obtained, and the core genes of PMZ in AD were screened. The intersection genes were enriched by Gene Ontology and Kyoto Encyclopedia of Genes and Genomes (KEGG) pathway enrichment analyses. In the in vitro experiment, mouse hippocampal neurons (HT22) were divided into control, glutamate (GLU) model, and GLU + PMZ treatment groups. The control group was given a complete culture medium, the model group was given GLU for 24 hours, the treatment group was given PMZ pretreatment for 3 hours, and then GLU was administered for 24 hours. Cell viability was determined, cell morphology was observed by microscopy, reactive oxygen species levels and glutathione content were detected, and protein expression of P53, PTGS2, SLC7A11, and GPX4 was detected by western blotting. Network pharmacology: A total of 317 PMZ targets, 1934 AD genes, 125 intersection genes, and 18 core genes, including P53 and PTGS2. Gene Ontology enrichment analysis showed that the effect of PMZ on AD was mainly related to cell proliferation, inflammation, hypoxia, synaptic structure, plasma membrane, and oxidoreductase activity. Kyoto Encyclopedia of Genes and Genomes results showed neuroactive ligand–receptor interaction, cell senescence, cancer pathway, PI3K-AKT signal pathway, neurodegeneration, and HIF-1 signal pathway. In vitro experiments: PMZ improved the GLU-induced decrease in cell viability and morphological changes in hippocampal neurons. PMZ inhibited reactive oxygen species levels and increased glutathione content in injured hippocampal neurons. Up-regulated of P53, SLC7A11 and GPX4 expression, and inhibited expression of PTGS2. PMZ regulates the SLC7A11–GPX4 antioxidant system to protect hippocampal neurons from oxidative stress injury.

## 1. Introduction

Alzheimer Disease (AD) is a degenerative disease of the central nervous system that mainly occurs in the elderly and is characterized by progressive cognitive dysfunction and behavioral damage.^[1]^ Its typical characteristics include extracellular amyloid Aβ peptide formation^[2]^ and intracellular hyperphosphorylated tau protein accumulation to form neurofibrillary tangles.^[3]^ AD is the most common type of dementia, accounting for 60% and 80% of dementia.^[4]^ At present, there are no drugs to delay the progression of the disease, and the search for drugs to treat AD has become a hot research direction.

Promethazine (PMZ) is a histamine H1 receptor antagonist and a phenothiazine drug that can induce sedation, relieve pain, and treat allergic reactions. In addition to these effects mentioned above, PMZ also shows neuroprotective by inhibiting neuroinflammation and oxidative stress in cerebral ischemia.^[5,6]^ In regulating apoptosis, PMZ can promote tumor cell apoptosis^[7,8]^ and also plays an antiapoptotic role in cerebral ischemia.^[9]^ Studies have shown that phenothiazines play a potential role in the treatment of neurodegenerative diseases,^[10]^ for example, methylene blue can improve the cognitive impairment in AD by promoting Aβ clearance, inhibiting tau protein phosphorylation and inhibiting neuroinflammation.^[11–13]^ Some studies have used a combination of high-throughput screening and MALDI imaging mass spectrometry and found that PMZ could bind to amyloid plaques.^[14,15]^ Previous studies have reported the antioxidant effect of PMZ on kidney injury and liver injury.^[16]^ However, there are few studies on the role of PMZ in AD; therefore, this study investigated the protective mechanism of PMZ against hippocampal neuronal injury.

## 2. Materials and methods

### 2.1. Network pharmacology

#### 2.1.1. Screening of PMZ and AD genes

‘Promethazine’ as the keyword, PMZ targets were searched in DrugBank, CTD, DGIdb, Genecards, PubChem, Stitch, and TTD databases. 2D chemical structure formula of PMZ was obtained through PubChem database and uploaded to Swiss Target Predication to obtain prediction targets. With “Alzheimer Disease” as the keyword, the AD genes were found in the GeneCards database. Relevance scores were used to sequence the results, and genes with scores >10 were selected as AD genes. Subsequently, the disease genes and drug targets were standardized using the UniProt database.

#### 2.1.2. Obtaining intersection gene for PMZ and AD

The selected drug targets and disease genes were uploaded to the Venny2.1. The name of the AD gene was input into list 1, and the name of the PMZ target was input into list 2 to obtain the intersection target of the PMZ and AD.

#### 2.1.3. Construction of protein–protein interaction network to screen AD core targets for PMZ interaction

The intersection targets were input into the STRING12 platform, Multiple Proteins were selected, and Homo Sapiens was defined. The PPI network between PMZ and AD was obtained with the highest confidence of 0.9. PPI information was imported into Cytoscape 3.9.1 software to draw protein interaction visualization and screen core genes. The top 10 core genes were screened according to betweenness centrality (BC), degree centrality (DC), and closeness centrality (CC).

#### 2.1.4. Kyoto Encyclopedia of Genes and Genomes pathway and Gene Ontology functional enrichment analysis to obtain the mechanism of PMZ on AD

The intersecting genes were uploaded to the DAVID online platform. KEGG pathway enrichment analysis and GO enrichment analysis, including biological processes (BP), molecular functions, and cellular components, were performed. The top 20 enrichment results were drawn into bar charts and bubble charts using bioinformatics online platform. Statistical significance was set at *P* < .05.

### 2.2. Vitro experiment

#### 2.2.1. Experimental cells

The cells are HT22, which are commonly used in the study of AD (Zhejiang Noble Biological Products Co., Ltd.).

#### 2.2.2. Reagents and instruments

Glutamate (GLU), and 3-(4,5-dimethyl-2-thiazolyl)-2,5-diphenyl-2-H-tetrazolium bromide (MTT) (Sigma, USA), PMZ (Macklin, Shanghai, China), reactive oxygen species (ROS) test kit, and glutathione (GSH) test kit (Beyotime, Shanghai, China). P53, PTGS2, GPX4, and SLC7A11 antibodies were purchased from Proteintech (USA). Microplate Reader (Bio-Rad, USA), and inverted fluorescence microscope (Leica, Germany).

#### 2.2.3. Cell culture

HT22 cells were cultured in a 10 cm diameter petri dish containing Dulbecco Modified Eagle medium (high glucose) + 10% fetal bovine serum + 1% penicillin–streptomycin double antibody and then placed in a 37 °C, 5% CO_2_ incubator.

#### 2.2.4. Cell grouping

The control group (Control) was given complete medium, the GLU model group (GLU) was given GLU 5 mmol/L for 24 hours, and the GLU + PMZ treatment group was pretreated with 0.5 μmol/L PMZ for 3 hours, followed by 5 mmol/L GLU for 24 hours.

#### 2.2.5. Determination of cell viability by MTT method

When the cells grew to 80% of the bottom area of the petri dish, they were digested with pancreatic enzymes, counted, and evenly spread into 96-well plates. When the cell density reached 70%, drugs were added (6 wells were set for each concentration). After 24 hours, the medium was discarded, and 20 μL MTT solution was added. The cells were cultured in an incubator for 3.5 hours, the MTT solution was discarded. 150 μL dimethyl sulfoxide was added to each well and then oscillated for 10 minutes until the crystallization was completely dissolved. A microplate reader was used to determine the absorbance at 490 nm.

#### 2.2.6. Observation of cell morphology

According to the grouping of cells, drugs were added, and the cell morphology was observed under a microscope.

#### 2.2.7. ROS detection

When HT22 cells in the petri dish grew to 80% of the dish bottom area, they were digested with pancreatic enzymes and counted, and 2 × 10^5^ cells per well were evenly spread a 6-well plate. When HT22 cells reached 70% of the dish bottom area, drug treatment was administered according to the above cell groups. The dichlorodihydrofluorescein diacetate probe was diluted in DEME high glucose medium (1:1000). The cell medium was removed, and diluted dichlorodihydrofluorescein diacetate was added to each well to cover all cells. The 6-well plate was wrapped in tin foil and incubated in an incubator for 20 minutes in the dark. The cells were washed 3 times with Dulbecco’s Modified Eagle’s Medium high-sugar medium and photographed using an inverted Lycra microscope. Image J software was used to quantify the average fluorescence intensity.

#### 2.2.8. Determination of GSH content

Standard products and samples were prepared according to the manufacturer’s instructions. After addition, mix well, incubate at 25 °C for 5 minutes, add 50 μL NADPH (0.5 mg/mL) solution into each well, and mix well. The absorbance value of A412 was immediately measured using a microplate Reader every 5 minutes for 25 minutes. A standard curve was drawn and the GSH content of the sample was calculated according to the standard curve. Another part of the cells was lysed, and bicinchoninic acid was used to determine the protein concentration. Relative GSH content was calculated and normalized.

#### 2.2.9. Western blot analysis

When the cells reached 70% of the bottom area, each group was administered drug treatment according to the cell group. After drug treatment, the culture medium in the petri dish was discarded, and 3 mL phosphate buffered solution was added to clean the culture dish 3 times. The lysate (phenylmethylsulfonyl fluoride:radio immunoprecipitation assay = 1:100) was added to the petri dish and allowed to crack on ice for 10 minutes. The supernatant was centrifuged, bicinchoninic acid protein quantification was performed according to the manufacturer’s instructions, and the prepared sample was denatured at 100 °C for 10 minutes. Protein bands were visualized by electrophoresis, membrane transfer, sealing, antibody incubation, and chemiluminescence. Protein bands were analyzed using the Image J software.

#### 2.2.10. Statistical analysis

Image J software was used for data quantification. GraphadPrism8.0.2 software was used for statistical analysis and presented as mean ± SEM. One-way ANOVA was used to compare the differences among the groups, followed by Tukey and Dunnett tests. The Value of *P* < .05 was considered statistically significant.

## 3. Results

### 3.1. Network pharmacological results

#### 3.1.1. PMZ and AD genes

**Figure 1.**
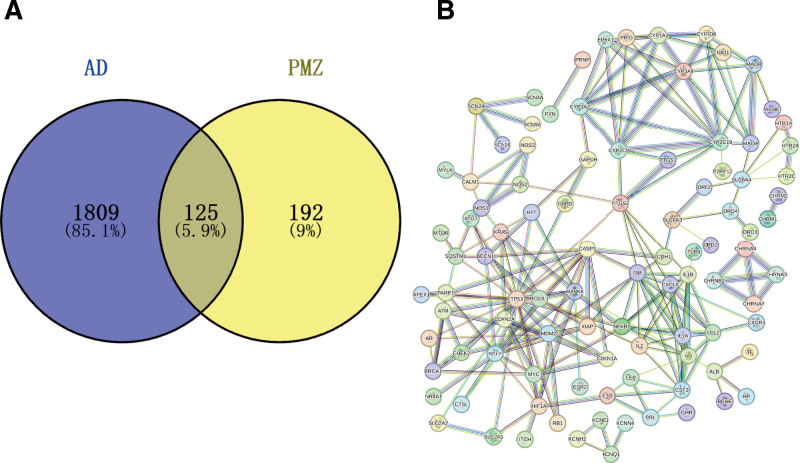
Venny diagram and protein–protein network interaction diagram. (A) 317 PMZ targets and 1934 AD genes were obtained through databases. 125 PMZ and AD intersection genes were obtained through the Venny 2.1.0 online website. (B) The 125 intersection genes were imported into the String platform to get the protein interaction map. The protein–protein interaction map contains 125 nodes, 221 edges, and 69 prediction edges.

A total of 317 PMZ targets were identified using the DrugBank, CTD, DGIdb, Genecards, PubChem, Stitch, and TTD databases and the Swiss platform. A total of 14,407 AD genes were found in the GeneCards database, and the screening condition was that the relevance score was >10. Finally of 1934 AD genes were identified. A total of 125 PMZ and AD intersection genes were obtained from the Venny 2.1.0 online website (Fig. 1A).

#### 3.1.2. Core genes of PMZ effect on AD

**Figure 2.**
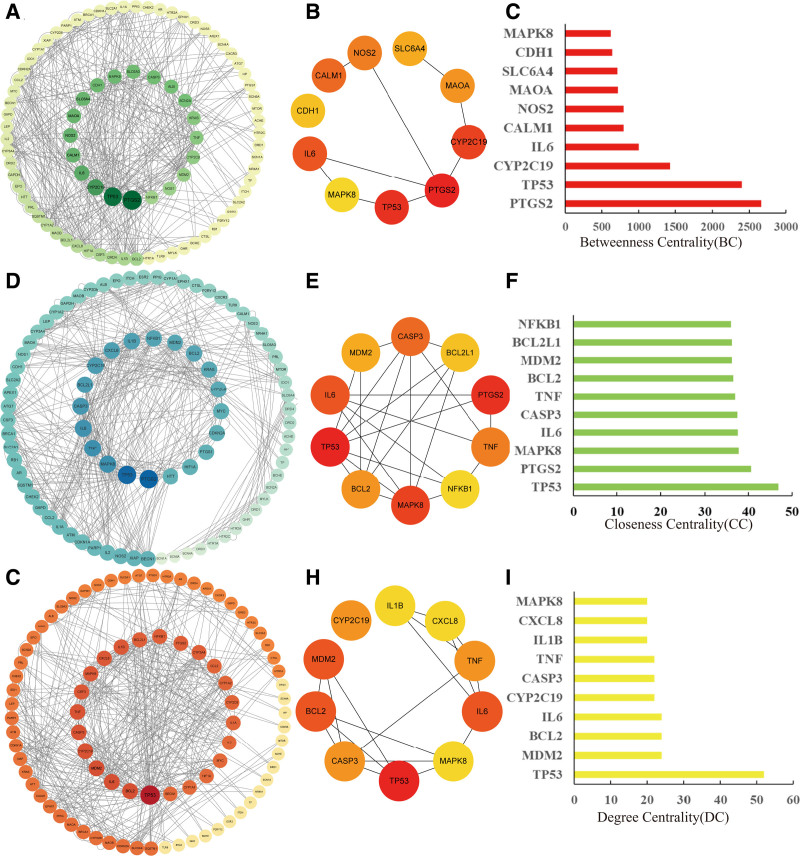
PPI network analysis and core genes screening by Cytoscape software. The larger the area of nodes in the figure and the darker the color, the more critical they are in the network (A, C) PPI network diagram of intersection genes arranged according to BC values and top 10 core genes (D, F) PPI network diagram of intersection genes arranged according to CC values and top 10 core genes. (G, I) PPI network diagram of intersection genes arranged according to DC values and top 10 core genes. BC = betweenness centrality, CC = closeness centrality, DC = degree centrality, PPI = protein–protein interactions.

The 125 intersection genes were imported into the String platform, and a high confidence of 0.9 was selected as the screening condition to obtain the protein interaction map. The protein interaction map contained 125 nodes, 221 edges, and 69 prediction edges (Fig. 1B). Protein interaction data were imported into Cytoscape3.9.1 software. The CytoNCA plug-in was installed to visualize protein–protein interactions according to the BC value (Fig. 2A), DC value (Fig. 2D), and CC value (Fig. 2G). The larger the area of the nodes in the figure and the darker the color, the more important they are in the network. The CytoHubba plug-in was used to screen the top 10 core genes. According to the BC value, the top 10 genes were PTGS2, TP53, CYP2C19, IL6, CALM1, NOS2, MAOA, SLC6A4, CDH1, and MAPK8 (Fig. 2B and C). According to the CC value, the top 10 genes were TP53, PTGS2, MAPK8, IL6, CASP3, TNF, BCL2, MDM2, BCL2L1, and NFKB1 (Fig. 2E and F). According to the DC value, the top 10 genes were TP53, MDM2, BCL2, IL6, CYP2C19, CASP3, TNF, IL1B, CXCL8, and MAPK8 (Fig. 2H and I). The darker the color in the image, the more the node protein interacts with the surrounding proteins.

#### 3.1.3. Mechanism of PMZ on AD

**Figure 3.**
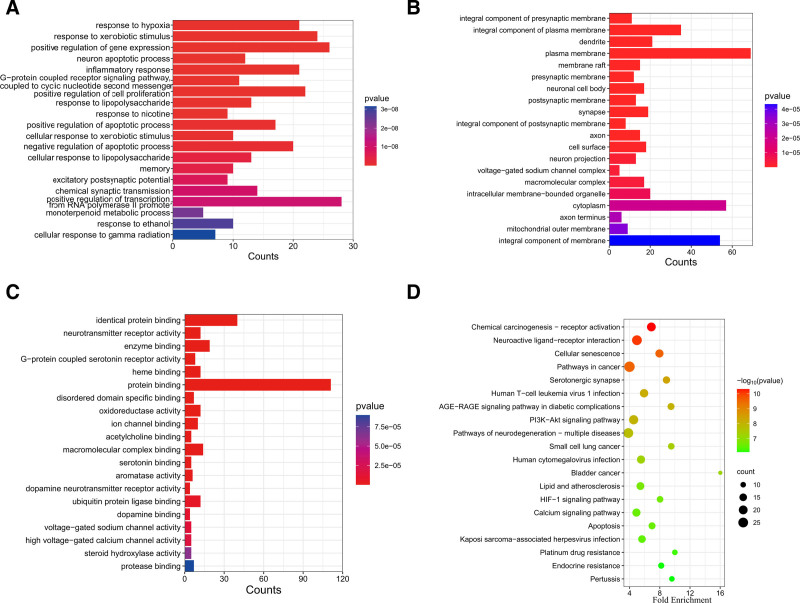
GO and KEGG enrichment analysis of intersection genes of PMZ and AD (top 20). (A) Biological process (BP). (B) Cell composition. (C) Molecular function (MF). (D) KEGG pathway enrichment analysis diagram (top 20). AD = Alzheimer disease, GO = Gene Ontology, KEGG = Kyoto Encyclopedia of Genes and Genomes, PMZ = promethazine.

The intersection genes of PMZ and AD were analyzed by GO and KEGG enrichment analysis using the DAVID database, and the top 20 items were visualized. There were 467 BP items in the GO enrichment analysis. It is mainly related to the hypoxia response, neuron apoptosis, inflammation, and regulation of cell proliferation in BP (Fig. 3A). There are 66 items of cellular component. This was mainly related to the neuronal structure, plasma membrane, and synaptic structure (Fig. 3B). There are 108 molecular functions items. It is mainly associated with a variety of neurotransmitters (5-hydroxytryptamine, acetylcholine and dopamine), oxidoreductase activity, and aromatase activity (Fig. 3C). A total of 124 KEGG signaling pathways were enriched, which were mainly related to neuroactive ligand–receptor interaction, cell aging, cancer pathway, PI3K-AKT signaling pathway, neurodegeneration, and the HIF-1 signaling pathway (Fig. 3D).

### 3.2. Results of in vitro experiment

#### 3.2.1. Determination of PMZ and GLU concentrations

**Figure 4.**
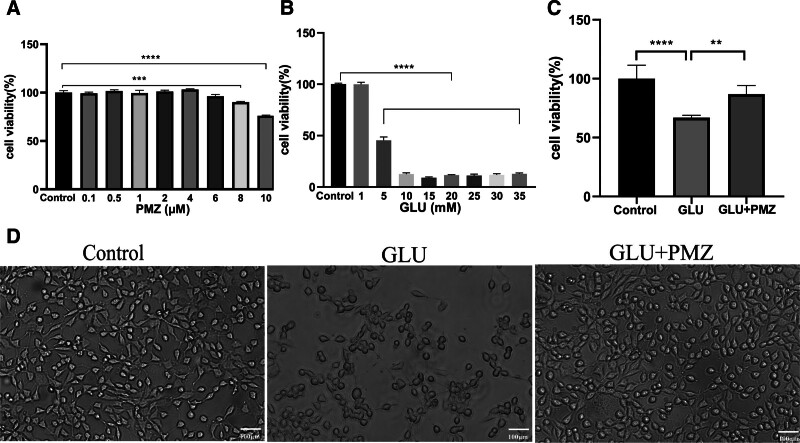
The effects of PMZ on cell viability and cell morphology induced by GLU. (A) Effects of different concentrations of PMZ on cell viability. 8 μM vs Control (***P < .001). 10 μM vs Control (****P < .0001). (B) Effects of different GLU concentrations on cell viability. ****P < .0001 vs Control. (C) The effect of PMZ on cell viability induced by GLU. GLU vs Control (****P < .0001), GLU vs GLU + PMZ (***P < .001). (D) Cell morphology under the microscope, scale bars 100 μm. GLU = glutamate, PMZ = promethazine.

The concentrations of 0.1, 0.5, 1, 2, 4, 6, 8, and 10 μM PMZ were selected. The results showed no significant change in cell viability at a PMZ concentration of 0.1 to 6 μM. Compared with the control group, the cell viability of 8 μM decreased by 9.9% (*P* < .001), and that of 10 μM decreased by 24% compared with the control group (*P* < .0001). Combined with the results and the literature,^[16]^ a concentration of 0.5 μM was selected as the therapeutic concentration of PMZ (Fig. 4A).

GLU is a common injury model in HT22 cells, and the concentration of GLU in the model is mainly in the range of 5 to 25 mM. Therefore, concentrations of GLU (1, 5, 10, 15, 20, 25, 30, and 35 mM) were selected for drug cytotoxicity exploration. It was found that the cell viability decreased by 55% (*P* < .0001) when the concentration was 5 mM compared with the control group. The concentration of 5 mM was selected as the model concentration (Fig. 4B).

#### 3.2.2. The effects of PMZ on cell viability and cell morphology of HT22 cells induced by GLU

Compared to the control group, cell viability in the GLU group decreased by 34%, and the difference was statistically significant (*P* < .0001); however, after 3 hours of PMZ pretreatment, cell viability increased by 20%, and the difference was statistically significant (Fig. 4C, *P* < .0001). These results indicated that PMZ could protect cells from injury caused by GLU.

Optical microscopy also observed cell morphology, showing that the number of cells decreased and became round after GLU injury. In contrast, PMZ pretreatment significantly increased the number of cells, and the cell shape became irregular with long spindles (Fig. 4D).

#### 3.2.3. Effect of PMZ on intracellular ROS induced by GLU

**Figure 5.**
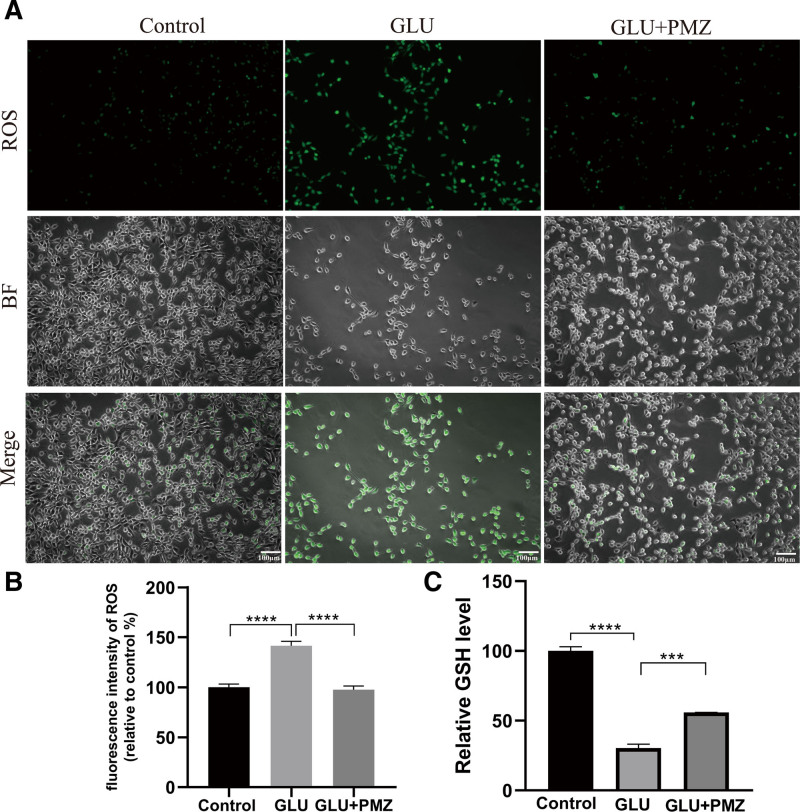
Effect of PMZ on intracellular ROS and GSH level induced by GLU. (A) The ROS was measured by DCFH-DA staining. The green staining represents ROS, scale bars 100 μm. (B) Results of statistical analysis of average fluorescence intensity of ROS. GLU vs Control (****P < .0001). GLU vs GLU + PMZ (****P < .0001). (C) Effect of PMZ on the change of GSH content in cells induced by GLU. GLU vs Control (****P < .0001). GLU vs GLU + PMZ (***P < .001). DCFH-DA = dichlorodihydrofluorescein diacetate, GLU = glutamate, GSH = glutathione, PMZ = promethazine, ROS = reactive oxygen species.

ROS represent oxidative damage. The average fluorescence intensity of the GLU group was significantly higher than that in the control group (*P* < .0001). However, the PMZ treatment was significantly lower than that of the GLU group (*P* < .0001). The results showed that PMZ reduced the GLU-induced increase in intracellular ROS (Fig. 5A and B).

#### 3.2.4. Effect of PMZ on the change of GSH content in cells induced by GLU

GSH is an important reducing agent. Compared to the control group, the GSH content in the GLU group decreased by 69.66% (*P* < .0001). After PMZ treatment, the GSH content increased by 25.55% compared to that in the GLU group (*P* < .001). The results indicated that PMZ partially alleviated the GLU-induced decrease in intracellular GSH content caused by GLU (Fig. 5C).

#### 3.2.5. Effect of PMZ on intracellular protein expression induced by GLU

**Figure 6.**
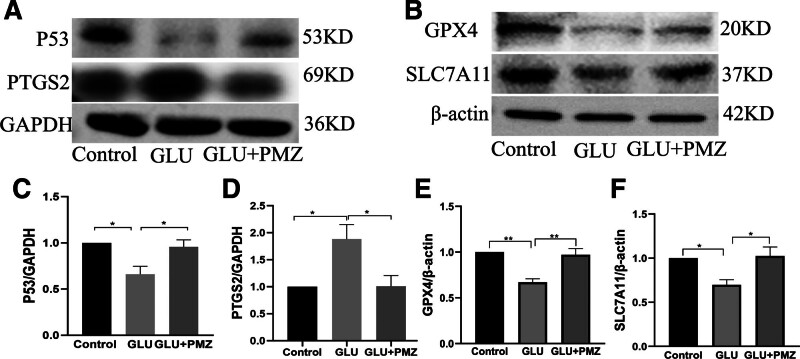
Effect of PMZ on intracellular protein expression induced by GLU. (A) Core gene P53 and PTGS2 protein expression. (B) Expression of antioxidant-related proteins. (C) Relative expression of P53, *P < .05. (D) Relative expression of PTGS2, *P < .05. (E) Relative expression of GPX4, **P < .05. (F) Relative expression of SLC7A11, *P < .05. GLU = glutamate, PMZ = promethazine.

Effects of PMZ on GLU-induced expression of P53, GPX4, SLC7A11, and PTGS2 in HT22 cells. Compared with the control group, the expression levels of P53 (*P* < .05), GPX4 (*P* < .01) and SLC7A11 (*P* < .05) of GLU group decreased, while the expression of PTGS2 increased (*P* < .05). Compared with GLU group, the P53 (*P* < .05), GPX4 (*P* < .01), and SLC7A11 (*P* < .05) expressions of PMZ group increased. In contrast, PTGS2 expression decreased (*P* < .05) (Fig. 6).

## 4. Conclusion

PMZ regulates the SLC7A11-GPX4 antioxidant system to protect hippocampal neurons from oxidative stress injury.

## 5. Discussion

AD is the most common type of dementia, with multiple coexisting mechanism. The primary mechanism hypothesis is the amyloid cascade reaction,^[17]^ hyperphosphorylation of tau protein,^[18]^ neurotransmitter disorder,^[19]^ neuroinflammation,^[20]^ vascular factor,^[21]^ and oxidative stress.^[22]^ Oxidative stress plays a vital role in different pathogenesis.^[23]^

Oxidative stress is oxidative damage caused by the accumulation of ROS due to an imbalance between the oxidative and antioxidant systems.^[24]^ Glutamate cystine reverse transporter (XC)-GPX4 is a vital antioxidant system. The XC-transporter consists of the 11subunit of the 7A member of the solute carrier family (SLC7A11 is also known as the cystine-glutamate transporter) and the SLC3A2 subunit (also known as 4F2HC), which is present on the phospholipid bilayer. It depends on ATP to cause cystine to be transported into the cell in exchange for glutamate, and cystine entering the cell is reduced to cysteine by GSH NAPDH or thioredoxin reductase 1.^[25]^ SLC7A11 determines the transport rate of the XC-transporter, and Se and GSH regulate the expression activity of GPX4. Glutathione is a tripeptide composed of glutamic acid, cysteine, and glycine, and is an important reducing agent, in which the sulfhydryl group of cysteine is its main functional group.^[26]^

To obtain more complete core genes, BC, CC, and DC were used for core gene screening. After removing duplicates,18 core genes were screened: TP53, MAPK8, IL6, PTGS2, CYP2C19, BCL2, CASP3, MDM2, TNF, CALM1, NOS2, MAOA, SLC6A4, CDH1, BCL2L1, NFKB1, IL1B, and CXCL8. These genes are closely related to the regulation of cell proliferation, apoptosis, inflammatory responses, and polyunsaturated fatty acid oxidation, etc. At the same time, the above genes are also present in the top-ranked GO enrichment analysis and KEGG pathway analysis. The expression of P53 and PTGS2 proteins at the core genes was verified using in vitro experiments.

GO enrichment analysis of the biological process, which included hypoxia, neuronal apoptosis, inflammation, and positive regulation of cell proliferation, ranked at the top. Hypoxia affects a variety of pathological processes in AD, including oxidative stress, mitochondrial dysfunction, inflammatory factor release, apoptosis, and other processes.^[27]^ Hypoxia and inflammation are interrelated; for example, hypoxia promotes the secretion of inflammatory factors, and the secretion of inflammatory factors will aggravate tissue hypoxia.^[28]^ An experimental study observed that the expression of hypoxia-inducing factors and inflammatory factors in AD mice was significantly increased, and the inflammatory proteins in cerebral vascular endothelial cells were elevated under hypoxia.^[29]^ Simultaneously, hypoxia-inducing factors promote apoptosis.^[27]^ Hypoxia also induces mitochondrial dysfunction and enhances ROS production and mitochondrial damage. In terms of molecular function, oxidoreductase activity is enriched, and monoamine oxidase A exists in the core gene and oxidoreductase activity gene. Using monoamine oxidase inhibitors in patients with AD can reduce the level of ROS in neurons, avoid oxidative damage, and improve cognitive impairment.^[30]^ The KEGG signal pathway includes neuroactive ligand-receptor interaction, cell senescence, cancer pathway, PI3K-AKT signal pathway, neurodegeneration, and HIF-1 signal pathway. These signal pathways are closely associated with AD.

HT22 cells are commonly used in the study of AD. These cells lack GLU receptors but are still sensitive to high extracellular GLU concentrations. A high concentration of extracellular GLU inhibits the glutamate cystine antiporter, decreasing intracellular GSH synthesis, ROS accumulation, and HT22 oxidative damage.^[31]^ PMZ is a drug with cytochrome P450 as a substrate that has antioxidant activity.^[16]^ However, currently, there is little research on AD. The cell viability and morphology results in this study showed that PMZ improved GLU-induced cell injury. The ROS detection results showed that PMZ inhibited the GLU-induced increase in ROS. The results of GSH content determination showed that PMZ reversed the decrease in GSH caused by GLU. Western blot results showed that PMZ up-regulated P53, SLC7A11, and GPX4 but inhibited the expression of PTGS2 to improve hippocampal neuronal damage induced by GLU. P53 is a common tumor suppressor and a redox-regulatory protein.^[32]^ P53 has a dual role in oxidative stress. When oxidative stimulation is weak, P53 promotes the expression of the target genes Sestrin, glutathione peroxidase, and aldehyde dehydrogenase while reducing the level of intracellular ROS, thus exerting an antioxidant effect. When oxidative stress level exceeds the antioxidant capacity of cells or the body, P53 promotes the expression of oxidative genes, inhibits the expression of antioxidant genes, increases the ROS production, and induces cell death or cell cycle arrest.^[33,34]^ PTGS2 (COX2) is a key enzyme that catalyzes the conversion of arachidonic acid into prostaglandins.^[35]^ Increasing evidence has shown that the expression of PTGS2 is related to the production of prostaglandins induced by oxidative stress. ROS can stimulate inflammatory responses, such as the expression of PTGS2. PTGS2 is an important source of ROS, and activation of the PTGS2 pathway may lead to oxidative stress.^[36]^ PTGS2 inhibitors reduce oxidative stress and inflammation.^[37]^ There is a relationship between PTGS2 and P53 expression. The activation of P53 has been shown to reduce the expression of PTGS2, thereby reducing the inflammatory response and oxidative stress.^[38]^

In summary, PMZ regulates the SLC7A11-GPX4 antioxidant system to protect hippocampal neurons from oxidative stress injury.

## Author contribution

**Data curation:** Li Bai.

**Methodology:** Li Bai.

**Software:** Li Bai.

**Supervision:** Fang Li.

**Validation:** Fang Li.

**Writing – original draft:** Li Bai.

**Writing – review & editing:** Fang Li.
